# Flat PVDF Membrane with Enhanced Hydrophobicity through Alkali Activation and Organofluorosilanisation for Dissolved Methane Recovery

**DOI:** 10.3390/membranes12040426

**Published:** 2022-04-15

**Authors:** Ramón Jiménez-Robles, Beatriz María Moreno-Torralbo, Jose David Badia, Vicente Martínez-Soria, Marta Izquierdo

**Affiliations:** 1 Research Group in Environmental Engineering (GI2AM), Department of Chemical Engineering, School of Engineering, University of Valencia, Avda. Universitat s/n, 46100 Burjassot, Spain; ramon.jimenez@uv.es (R.J.-R.); vmsoria@uv.es (V.M.-S.); 2 Research Group in Materials Technology and Sustainability (MATS), Department of Chemical Engineering, School of Engineering, University of Valencia, Avda. Universitat s/n, 46100 Burjassot, Spain; bmmortor@itq.upv.es (B.M.M.-T.); jose.badia@uv.es (J.D.B.)

**Keywords:** anaerobic digestion, hydrophobicity, membrane stability, methane recovery, organofluorosilane, PVDF, surface modification

## Abstract

A three-step surface modification consisting of activation with NaOH, functionalisation with a silica precursor and organofluorosilane mixture (FSi_T_), and curing was applied to a poly(vinylidene fluoride) (PVDF) membrane for the recovery of dissolved methane (D-CH_4_) from aqueous streams. Based on the results of a statistical experimental design, the main variables affecting the water contact angle (WCA) were the NaOH concentration and the FSi_T_ ratio and concentration used. The maximum WCA of the modified PVDF (mPVDF_max_) was >140° at a NaOH concentration of 5%, an FSi_T_ ratio of 0.55 and an FSi_T_ concentration of 7.2%. The presence of clusters and a lower surface porosity of mPVDF was detected by FESEM analysis. In long-term stability tests with deionised water at 21 L h^−1^, the WCA of the mPVDF decreased rapidly to around 105°, similar to that of pristine nmPVDF. In contrast, the WCA of the mPVDF was always higher than that of nmPVDF in long-term operation with an anaerobic effluent at 3.5 L h^−1^ and showed greater mechanical stability, since water breakthrough was detected only with the nmPVDF membrane. D-CH_4_ degassing tests showed that the increase in hydrophobicity induced by the modification procedure increased the D-CH_4_ removal efficiency but seemed to promote fouling.

## 1. Introduction

Methane (CH_4_) produced as biogas in anaerobic digesters is partially dissolved in the digestate, so the anaerobic reactor effluents can present high amounts of dissolved CH_4_ (D-CH_4_), the discharge of which causes fugitive emissions of this important greenhouse gas and biofuel. The use of membrane contactors as desorption devices for the recovery of D-CH_4_ from anaerobic reactor effluents has attracted the strong interest of researchers in recent years [1,2,3,4,5]. As well as CH_4_ degassing from aqueous streams, membrane contactors can also be used in other stripping and gas absorption processes such as CO_2_ capture from flue gas or biogas streams or gas removal from aqueous solutions using transmembrane chemical absorption [6,7]. The most common industrial configuration is the hollow-fibre membrane contactor (HFMC), characterised by a high specific surface area, compactness, ease of scaling, and cost-effectiveness. Nevertheless, HFMC is easy to damage and susceptible to pore blocking owing to the stress from the liquid flux and/or the fouling phenomenon [6,8,9], and it cannot be easily repaired or refreshed which can dramatically increase the maintenance and operation costs. Although many studies have focused on HFMCs, flat-sheet membrane modules (FMs) present several advantages related to their simplicity, versatility, and ease of cleaning and replacement, which make them attractive for use in some applications and also in investigations at a laboratory scale [8]. FMs can be especially useful where fouling or membrane stability are the focus of the research, or to facilitate the performance comparison of membrane materials with different characteristics [8,10]. The most typically used polymers for commercial membrane contactors include polydimethylsiloxane (PDMS), polysulfone (PSF), polyacrylonitrile (PAN), polypropylene (PP), polytetrafluoroethylene (PTFE), and poly(vinylidene fluoride) (PVDF). PVDF membranes have been intensively studied in recent years [11] due to their high performance and thermal, mechanical, and chemical resistance [5,12]. In addition, they present a lower raw material cost and ease of synthesis, casting, and extrusion to form different membrane configurations [12] as well as having a good thermodynamic affinity with various fluoropolymers [13].

Despite the numerous advantages of polymeric membrane contactors relative to traditional separation units, two major shortcomings are apparent when operating with membranes: wetting and fouling phenomena. In microporous membranes, the liquid pressure over the membrane surface can induce the partial or total filling of the membrane pores with the liquid phase when a critical transmembrane pressure is surpassed [5,14]. This membrane wetting increases the mass transfer resistance and, consequently, reduces the separation efficiency. Only 5% of membrane pores occupied by the liquid phase can induce an increase of around 20% in the overall mass transfer resistance [15]. The wetting resistance is influenced by membrane properties such as the pore size and distribution, roughness and, especially, the hydrophobicity of the membrane material [5,16,17]. In operation with anaerobic reactor effluents, the fouling agents contained in the stream can adhere to the membrane surface [18]. This fouling cake adds restrictions for mass transfer and can also physically and chemically deteriorate the membrane [9]. The tendency of the membrane to be fouled depends mainly on issues such as membrane hydrophobicity and roughness, the concentration of the foulants and ionic strength of the solution, and membrane module configuration, among other factors [9,18,19].

In order to overcome these shortcomings, polymer membranes can be enhanced at the synthesis step by controlling different operational conditions or by using additives to add new functionalities or change the membrane morphology [9,20]. The surface modification of existing membranes has gained a great deal of attention in recent years due to the versatility offered by the different techniques [9,12]. Surface modification techniques can be divided into physical and chemical treatments. Chemical treatments commonly involve grafting modifying agents onto the membrane by means of covalent bonding, thus achieving a stronger adhesion force. Consequently, the modifiers are less susceptible to removal from the membrane by liquid flux. Recently, surface chemical modification has focused on increasing the hydrophobicity of the membrane surface to avoid the wetting phenomenon [10,21,22]. Thus, fluorinated organic compounds and siloxanes have been developed and evaluated as modifying agents, since they are well-known to reduce surface free energy and, consequently, to increase hydrophobicity [5]. Indeed, the superhydrophobic degree (water contact angle, WCA > 150°) has been reported after surface modification with organosilanes [23]. In the case of PVDF, PTFE, or PDMS, with poor reactivity, a step prior to grafting the modifiers is needed to activate the membranes [5,12]. Different techniques, such as hydroxylation by NaOH or plasma treatment, have been evaluated, in which the membrane is subjected to an alkaline or oxygen-radical environment, respectively, in order to form active sites on the membrane (e.g., hydroxide or peroxide groups) to react further with the functionalisation agents that confer enhanced surface performance [17,24,25].

The long-term stability of the membrane during operation plays a critical role in the design of the surface modification [26]. The stress and dragging effect of a continuous liquid flux over the modified membranes in long-term operations can remove the coating in the case of physical modification or alter the surface morphology, producing a change in the surface properties such as the hydrophobicity [8,19,27] and reducing the membrane lifetime. Reports into the evaluation of the structural and chemical stability of modified membranes after long-term operations for D-CH_4_ recovery are very scarce but would provide useful preview information about the economic viability of applying membrane modification procedures at the industrial scale. The absorption of CO_2_ using membrane contactors has been more widely investigated, and higher CO_2_ fluxes and stability in long-term tests have been reported with modified membranes compared to non-modified membranes under different modification procedures [25,28,29,30,31].

In this context, the general aim of this work was to evaluate the effect of a chemical surface modification applied over a commercial PVDF membrane for the recovery of D-CH_4_ from aqueous streams. Firstly, a statistical experimental design of a three-step modification procedure (activation, functionalisation, and curing) was proposed to optimise the surface hydrophobicity of the membrane, measured by the WCA. Then, the stability and the membrane performance in D-CH_4_ recovery by modified PVDF membranes were evaluated and benchmarked with non-modified PVDF. These membranes were tested in an FM module under several operational conditions with deionised water (DW) and real anaerobic reactor effluent (AE).

## 2. Materials and Methods

### 2.1. Membrane Surface Modification Procedure

Flat-sheet PVDF membranes were supplied by Dorsan Filtration (Spain). The membrane was composed of hydrophobic PVDF supported on a polyester (PET) non-woven support, resulting in a microporous structure. The main characteristics of the membrane are reported in Table 1.

The surface modification of the PVDF was carried out in a three-step procedure: (1) activation, (2) functionalisation, and (3) curing.

In the activation step, the PVDF was hydroxylated with 2.5 mL of sodium hydroxide (NaOH) solution per cm^2^ of membrane in an orbital shaker at 150 rpm. According to preliminary experiments (Supplementary Material S1), the temperature and time of the activation step were fixed at 50 °C and 1 h, respectively, and the NaOH concentration (NaOH%_wt_) ranged from 1 to 6%, as suitable for the purposes of activation. Afterwards, the activated membranes were immediately rinsed with milliQ water to stop the reaction and the excess of water on the sample surface was gently removed. In the hydroxylation reaction, the OH^−^ ions replaced the F atoms from the PVDF chains, which promoted a decrease in its hydrophobicity, mainly by a defluorination mechanism [24]. The detailed reactions proposed for the modification of PVDF are shown in the Supplementary Material S2.

In the functionalisation step, the organofluorosilane 1H,1H,2H,2H-perfluorooctyltriethoxysilane (Dyn; Dynasylan^®^ F8261, Evonik GmbH, Hanau-Wolfgang, Germany) and tetraethyl orthosilicate (TEOS; ≥99%, Sigma-Aldrich, Saint Louis, MI, USA) were used as modifying agent and silica precursor, respectively, to carry out the grafting of additional fluoride chains onto the PVDF. A mixture of 2-propanol (IPA; HPLC grade, VWR Chemicals, Radnor, PA, USA) and milliQ water in a molar ratio of 57:1 was used as solvent (labelled as IPA/H_2_O). For the preparation of the functionalisation solution (FS), an initial mixture of the organofluorosilane and silica precursor was prepared and then the IPA/H_2_O solvent was added dropwise [27]. The presence of water in the FS was needed in order to hydrolyse the silica precursor, as reported by several authors [27,31,33,34]. Once the solvent was fully added, in order to promote the hydrolysis of the TEOS [27,34], the pH of the FS was adjusted with concentrated HCl (37 wt %, reagent grade, VWR Chemicals, Radnor, PA, USA) to a value of 2–3. Finally, the FS remained under magnetic stirring at 200 rpm at room temperature for 24 h in order to achieve the complete hydrolysis of the TEOS. The mixture of Dyn and TEOS in the FS was labelled ‘FSi_T_’, where ‘F’ denotes the fluorine modifying agent Dyn and ‘Si_T_’ the silica precursor TEOS. The volumetric ratio of the modifying agent in the FSi_T_ mixture (FSi_T_ ratio = Dyn volume/FSi_T_ mixture volume) ranged from 0.17 to 0.94, and the FSi_T_ volumetric percentage in the solvent (FSi_T_%_v_) ranged from 0.8 to 9.2%. The ranges of both parameters were set from the preliminary experiments shown in Supplementary Material S1. In the functionalisation step, the membrane samples were immersed in 2 mL of the FS per cm^2^ of the membrane for 1 h at 100 rpm and room temperature and then rinsed with IPA. The reactions involved in the functionalisation are detailed in Supplementary Material S2.

**Figure 1:**
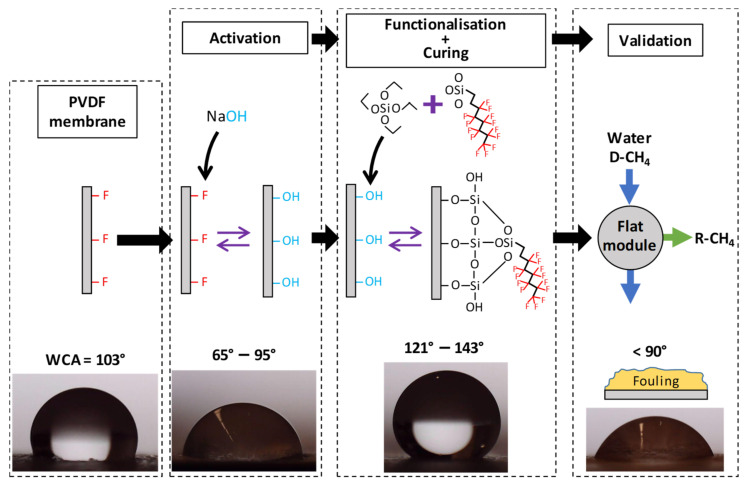
Scheme of the modification procedure of poly(vinylidene fluoride) (PVDF) membranes and the validation step. Water contact angle (WCA): static water contact angle; D-CH_4_: methane dissolved in liquid streams; R-CH_4_: methane recovered in gaseous form.

Finally, for the curing step, the membrane was kept in an oven at 60 °C overnight on a Petri dish. Functionalisation and curing times were also established based on the preliminary experiments. The modified PVDF was labelled ‘mPVDF’ and the non-modified PVDF as ‘nmPVDF’. A scheme of the membrane modification procedure is shown in Figure 1.

### 2.2. Design of Experiments and Statistical Analysis

The effect of the modification procedure on the hydrophobicity of PVDF was initially evaluated on 2 × 2-cm membrane specimens. The main variables involved in the modification procedure were analysed by means of a statistical experimental design in order to maximise the static WCA of the membrane surface. After preliminary experiments in which the activation time (1 h) and temperature (50 °C), and functionalisation and curing times (1 h and >12 h, respectively) were fixed, the main variables affecting the final hydrophobicity of the PVDF were attributed to FSi_T_ ratio, FSi_T_%_v_, and NaOH%_wt_. These variables (factors) were evaluated at the values (levels) shown in Table 2. A 3^3^ complete factorial design with the central point and corner levels was carried out in order to identify the significant effects of the factors and their interactions on the response variable, i.e., the WCA. Then, statistical analysis based on multiple linear regression and a response surface was carried out for the determination of the optimum values of the factors leading to the maximum response. This analysis included the additional axial points specified in Table 2. The different runs of the set of experiments were carried out randomly in duplicate to avoid systematic errors. The statistical software Minitab^®^ (Minitab, LLC., State College, PA, USA) was used for the design of the experiments and the statistical analysis.

### 2.3. Evaluation of the Modified Membrane Performance

The performance of recovery of D-CH_4_ from liquid streams and the long-term stability of both nmPVDF and mPVDF were evaluated in degassing tests and long-term tests, respectively. Experiments were carried out using DW (<1 µS cm^−1^) and an effluent stream from an anaerobic digester for comparison purposes. The anaerobic effluent (AE) was collected from the anaerobic reactor of the urban Quart-Benager II wastewater treatment plant (Valencia, Spain) and filtered with a 10–20 µm filter before use in the tests. The pH, conductivity, alkalinity, chemical oxygen demand, and total and volatile suspended solids of the filtered AE were 7.84, 8.61 mS cm^−1^, 2102 mg CaCO_3_ L^−1^, 414 mg O_2_ L^−1^, 75.75 mg L^−1^, and 67.50 mg L^−1^, respectively. A circular FM made of stainless steel with an effective contact area of 17.3 cm^2^ was used for both performance and long-term stability tests.

#### 2.3.1. Dissolved Methane Recovery from Water Streams

**Figure 2:**
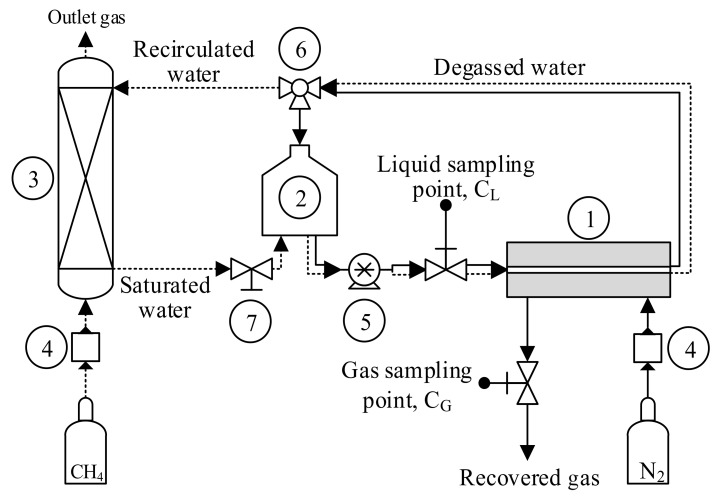
Scheme of the experimental system for dissolved methane degassing tests from aqueous streams with a flat-sheet membrane module. (1) membrane module, (2) liquid feed tank, (3) saturation column, (4) mass flow controller, (5) peristaltic pump, (6) 3-way valve, and (7) on/off valve.

The laboratory-scale system with the FM used for the non-steady-state degassing tests was extensively described in our previous work [8] and is depicted in Figure 2. For the degassing test, a 2 L glass bottle acting as the liquid feed tank was filled initially with the liquid (DW or AE). In the first step, the liquid was saturated with 99.5%_v_ CH_4_ (Carburos Metálicos, Barcelona, Spain) in a packed-bed absorption column in counter-current flux. The liquid stream flowed in a closed loop through the system using a peristaltic pump (Watson-Marlow Fluid Technology Solutions, Falmouth, UK), keeping the gas ports of the module closed (dashed line, Figure 2). This liquid stream reached a concentration of 20.7 ± 1.1 mg CH_4_ L^−1^. In the second step, the saturated liquid was pumped from the liquid feed tank to the FM and then recirculated continuously to the tank operating in a closed loop in order to recover the D-CH_4_ (continuous line, Figure 2). The total duration of these non-steady-state experiments was 5 h, and the liquid flow rates (Q_L_) were fixed at 3.5 and 21.0 L h^−1^ (liquid velocity ranging from 2.8 to 16.9 cm s^−1^) based on our previous work [8]. All experiments were carried out in the sweep-gas mode, using a N_2_ flow rate (Q_N2_) of 4 L h^−1^ (gas velocity of 0.3 cm s^−1^). The liquid and sweep gas flowed in a cross-flow configuration inside the FM and the experiments were conducted at room temperature (24 ± 1 °C). Removal of D-CH_4_ was evaluated from liquid samples of 5 mL collected at different times during the degassing test to determine the change in D-CH_4_ concentration measured by gas chromatography. Duplicates of liquid samples were collected at the outlet of the liquid feed tank. The results reported in this paper are the removal efficiency (RE, %) and the CH_4_ flux (J_CH4_) obtained after 5 h of experimentation. As an example, the profiles of the D-CH_4_ concentration, RE, and J_CH4_ obtained at a Q_L_ of 21 L h^−1^ are shown in Supplementary Material S3.

#### 2.3.2. Long-Term Surface Stability Tests

Experiments using DW and AE were carried out to evaluate the behaviour of the PVDF membranes during long-term operation using the former laboratory-scale system and FM. The long-term tests were carried out to evaluate the stability of the hydrophobicity of the membrane surface under a continuous liquid flux with different types of aqueous stream. Initially, the liquid feed tank was filled with DW or AE, the saturation column disconnected, and the gas ports of the FM closed for this type of test. Then, a constant water flux was applied through the liquid side of the FM in the closed loop defined by the continuous line in Figure 2. Based on our previous study [8], the long-term tests with DW were operated at the highest value of Q_L_ of 21 L h^−1^ until a constant WCA was reached. The long-term tests with AE were carried out at the lowest Q_L_ tested, 3.5 L h^−1^, in order to promote the potential fouling deposition on the membrane for up to around 830 h. In the experiments with AE, the anaerobic water inside the system was replaced up to three times. The membrane was extracted periodically to measure its WCA in order to monitor the surface hydrophobicity with time of use, as reported in the literature [8,27,33]. Similarly, for the membranes treating the AE, the membrane performance for D-CH_4_ degasification was evaluated at different times of use using AE saturated with CH_4_ as the liquid stream.

### 2.4. Analytical Methods

The surface hydrophobicity of the membranes was evaluated by means of the WCA as mentioned above. WCA measurements were conducted by the sessile drop technique [35] of depositing a water drop of 5.5 ± 0.1 μL onto the membrane surface using a syringe pump (KF Technology s.r.l., Rome, Italy) at room temperature (~25 °C). An image of the water drop profile was captured at 15 s with a digital microscope (Handheld Digital Microscope Pro, Celestron LLC., Torrance, CA, USA) under white light (Philips HUE Lamp, Koninklijke Philips NV, Amsterdam, The Netherlands). ImageJ software was used for image processing using the *Contact Angle Plug-in* based on the ellipse approximation. The WCA was evaluated at different positions on the membrane and a mean value obtained from at least four measurements. The WCA values of the wet membranes from the long-term stability tests were measured after removing excess water and evaporation of moisture at room temperature for approximately 1 h with forced aeration.

The morphology of the membrane surface and cross-section, as well as its chemical composition, were analysed by field emission scanning electron microscopy (FESEM) equipped with energy dispersive X-ray spectrometer (EDX) with an accelerating voltage of 20 kV (Hitachi S4800, Hitachi Ltd., Tokyo, Japan). For image acquisition, the samples were placed on a metal holder and then coated with a fine layer of Au/Pd by sputtering in vacuum for 1 min. FESEM was also applied to measure the membrane thickness and the surface porosity by means of the abovementioned ImageJ software [36].

The liquid samples collected from the degassing tests were analysed by the adapted head-space method described elsewhere [37,38,39,40]. Briefly, 5 mL liquid samples were collected in sealed 16 mL vials prefilled with air. These vials were allowed to reach equilibrium between phases in an orbital shaker at 200 rpm and 25 °C for 30 min. After this, 0.5 mL of head-space gas was injected into a gas chromatograph (GC 7820 A, Agilent Technologies Spain S.L., Madrid, Spain) equipped with Agilent HP-PLOT/U and Agilent HP-MOLESIEVE columns and a thermal conductivity detector to measure the CH_4_ concentration in the gas phase. The CH_4_ concentration in the liquid phase was calculated by applying Henry’s Law, and then both the gas and liquid phase concentrations were used to calculate the concentration in the liquid sample (C_L_, mg L^−1^).

The RE of D-CH_4_ (%) and CH_4_ flux (J_CH4_, g s^−1^ m^−2^) through the membrane were used to evaluate the performance of the D-CH_4_ recovery process in the degassing tests, defined as follows:

(1) $$\mathrm{RE}=\frac{C_{L0}-C_{\mathrm{Lt}}}{C_{L0}}\times 100$$

(2) $$J_{\mathrm{CH}4}=\frac{V_{T}\times 10}{A}\times \frac{{\mathrm{dC}}_{L}}{\mathrm{dt}}$$

where C_L0_ and C_Lt_ (mg L^−1^) are the CH_4_ concentrations in the liquid at the beginning and at time on stream (t) of the degassing test from the liquid feed tank, respectively; V_T_ is the total liquid volume in the system (2.35 L); and A (cm^2^) is the effective membrane area. The variation in liquid volume flow rate was assumed to be negligible for the application of Equation (1).

## 3. Results and Discussion

### 3.1. Maximisation of Membrane Hydrophobicity

**Figure 3:**
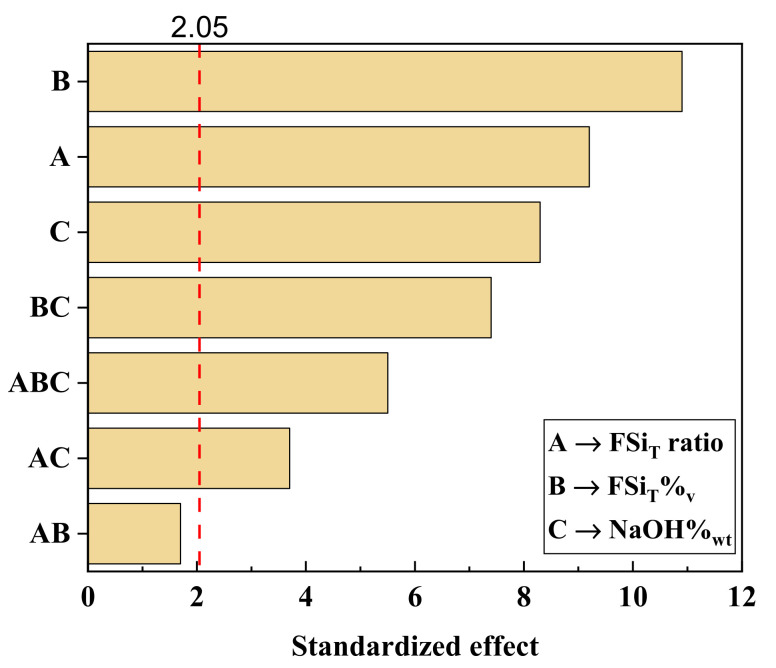
Pareto diagram of the standardised effects of the factors analysed in the 3^3^ complete factorial design. The dashed red line represents the critical standardised effect for a significance level of 0.05.

A statistical experimental design evaluating the main parameters that affect the membrane surface hydrophobicity was conducted to maximise the response variable, i.e., the WCA, of the modified membranes. After the preliminary experiments (Supplementary Material S1), the variables FSi_T_ ratio, FSi_T_%_v_, and NaOH%_wt_ were found to be the main factors to be optimised. Initially, a 3^3^ complete factorial design was carried out in order to determine the effects of the different factors, including individual effects and those of the 2-way and 3-way interactions on the response. The effects of the factors and their interactions can be observed in the Pareto diagram of the factorial design shown in Figure 3. The critical standardised effect (2.05) was calculated from the Student’s T distribution (t_α/2_,ν) with a significance level (α) of 0.05 and 27 degrees of freedom (ν), associated with the error of the factorial design. As observed in the Pareto diagram, the standardised effect of the different factors and their interactions were higher than the critical value, except for the interaction between FSi_T_ ratio and FSi_T_%_v_. Thus, the interaction of these two factors did not seem to affect the WCA of the mPVDF and their effects could be ignored.

The individual factors presented the highest effects on the response, the most important being those involved in the functionalisation step (FSi_T_ ratio and FSi_T_%_v_). This result can be understood, since the FSi_T_ ratio and FSi_T_%_v_ determined the number of O–Si bonds on the PVDF chains and the total F content at the end of the functionalisation process, these resulting in higher WCA values as the F content on the surface increased. Additionally, the increase in O–Si bonds grafted onto the PVDF chains allowed reaction with higher numbers of Dyn molecules, increasing the total F content of the surface of the modified membrane. The NaOH%_wt_ also showed a high effect on the response, since this factor determined the number of active sites (OH^−^) on the PVDF chains where the silanol groups from TEOS were grafted, leading to O–Si bonds [23,25]. Consequently, the higher the NaOH%_wt_ applied, the higher the number of F atoms from the PVDF chains that were substituted by OH^−^ groups, allowing a higher number of O–Si bonds and Dyn molecules to be grafted. This was in accordance with the significant standardised effect of the interaction of FSi_T_ ratio and FSi_T_%_v_ with NaOH%_wt_ (ABC) observed in the Pareto diagram.

**Figure 4:**
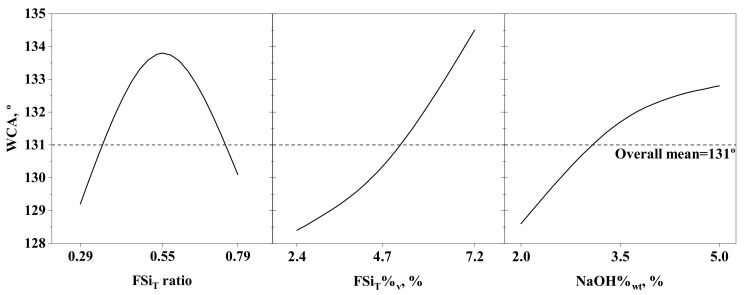
Main-effects plot of the factors analysed in the 3^3^ complete factorial design.

The effects of the individual factors on the response are depicted in the main-effects plot shown in Figure 4. A WCA higher than that of the nmPVDF (103.4 ± 1.6°) was obtained for all the modified membranes used, with an overall mean value of 131°, showing that the functionalisation procedure proposed in this work was suitable to increase the PVDF membrane hydrophobicity. The WCA clearly increased with the FSi_T_%_v_ and NaOH%_wt_, in accordance with the previous discussion, although NaOH%_wt_ should not be higher than 5% in order to avoid membrane degradation. In contrast, a maximum WCA was found with the variation of the FSi_T_ ratio, as reported by other authors [23,31]. In that case, the WCA increased initially with the FSi_T_ ratio until a maximum value was reached at a FSi_T_ ratio of around 0.55. An increase in FSi_T_ ratio caused a higher number of Dyn molecules to be grafted onto the PVDF chains. In contrast, a higher FSi_T_ led to a decrease in WCA, which could be explained by taking into account that the amount of TEOS decreased as Dyn increased, and therefore fewer O–Si bonds were formed for the subsequent grafting of Dyn molecules. Thus, a value of around 0.55 seemed to provide an appropriate number of O-Si bonds to graft the available Dyn onto the FS and avoid a large excess of unreacted Dyn.

**Figure 5:**
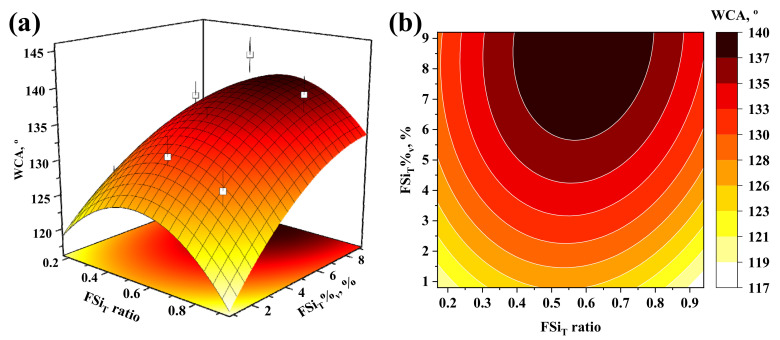
(**a**) Response surface and (**b**) contour plot obtained by linear regression from the overall data of the design of the experiments with a NaOH concentration of 5%. Experimental values are depicted as open symbols in the response surface plot and the error bars denote the standard deviation.

After determining the main effects of the factors and their interactions with the 3^3^ factorial design, a new set of experiments was conducted at the axis levels of the three factors. A statistical analysis based on response surface methodology was carried out on all the data obtained at the central point, corner, and axis levels. All these data were fitted by means of a multiple linear regression model including the linear (A, B, C), square (AA, BB, CC), 2-way interaction (AB, AC, BC), and 3-way interaction (ABC) terms. The 2-way interaction between FSi_T_ ratio and FSi_T_%_v_ was also included in the model. Since large effects were observed for the individual factors (A, B, and C, Figure 3), their square terms could also present high significance in the model and were therefore included. The resulting model is represented in the form of a response surface and contour plot at a NaOH%_wt_ of 5% in Figure 5.

Based on the model explained above, the WCA initially increased with FSi_T_%_v_ up to a value around 6% and then remained nearly constant for a fixed FSi_T_ ratio. For example, the model predicted a WCA of 137–140° for FSi_T_%_v_ ranging from 6 to 9.2% at a constant FSi_T_ ratio of 0.55 with a level of confidence of 95%. Regarding the effect of the FSi_T_ ratio on the WCA, the predicted trend showed a maximum value of WCA at intermediate values of FSi_T_ ratio, as reported in the literature [23,31]. Thus, the maximum WCA values were achieved for a FSi_T_ ratio of around 0.55 at any FSi_T_%_v_ value. This trend was in accordance with the previous analysis of effects and the experimental results shown in Figure 5a (open symbols). Thus, the maximum experimental WCA obtained was 143.2 ± 2.5°, while the model predicted a WCA of 138.8 ± 2.2° at an FSi_T_ ratio of 0.55, FSi_T_%_v_ of 7.2%, and NaOH%_wt_ of 5%. Likewise, the maximum WCA value predicted by the model was 140.5 ± 6.6° at an FSi_T_ ratio of 0.59, FSi_T_%_v_ of 9.2%, and NaOH%_wt_ of 6%. These results led to the conclusion that increases in FSi_T_ ratio, FSi_T_%_v_, and NaOH%_wt_ over 0.55, 7.2%, and 5%, respectively, would not significantly increase the WCA and that they could be set as the optimal experimental conditions to achieve the highest hydrophobicity. The PVDF sample modified under the optimal conditions is termed hereafter as ‘mPVDF_max_’.

For the sake of benchmarking the results, the maximum WCA obtained in this work (143.2 ± 2.5°) represented a 39% increase from the initial value of the nmPVDF (103.4 ± 1.6°) and was higher than most values found in the literature for comparable surface modification approaches. The maximum WCA was obtained at a low NaOH%_wt_ (5%) with IPA as solvent, unlike the other hazardous solvents such as toluene, hexane and tetrahydrofuran reported in the literature. Similar results of WCA increase have been reported by other authors. Sairiam et al. [25] reported a maximum WCA of 119.5° (from an initial value of 68.9°) with the same modifying agent as used in this work, using hexane as solvent. They applied longer treatments, and no WCA increase for the modified membranes was reported with NaOH%_wt_ higher than 10%. Wongchitphimon et al. [31] found a maximum WCA of 127.8° for modified PVDF (from an initial value of 95.5°) at a NaOH%_wt_, activation time, and temperature of 10%, 1 h, and 60 °C, respectively, and functionalised with the commercial organofluorosilane Fluorolink^®^ S10 (FS10) at a FSi_T_ concentration of 5% and a ratio of FS10 to TEOS of 3:2 using toluene as solvent. In addition, they suggested destruction of the membrane integrity, since they reported a final WCA decrease and an increase in pore size at activation times higher than 1 h and a NaOH%_wt_ of 10%. Moreover, Sethunga et al. [21] obtained a modified PVDF with a maximum WCA of 111.7° (from an initial value of 84.5°) with a somewhat lower NaOH%_wt_ (pH = 10) and activation and functionalisation times of 0.5 and 1.5 h using Fluorolink^®^ S10 and toluene as modifying agent and solvent, respectively. They also reported a final WCA decrease with an increase in NaOH%_wt_ and activation time, suggesting a conversion of the hydroxide groups to carbonyl groups that hindered the attachment of TEOS [21]. The maximum value was reported by Zheng et al. [23], with a maximum WCA of 157° (from an initial value of 89°) under more severe activation conditions with a NaOH%_wt_ of 30% and an activation time of 3 h and using organosilanes free of fluorine groups at a concentration of around 25% in the FS and toluene as solvent.

### 3.2. Structure and Chemical Composition of the Modified PVDF

**Figure 6:**
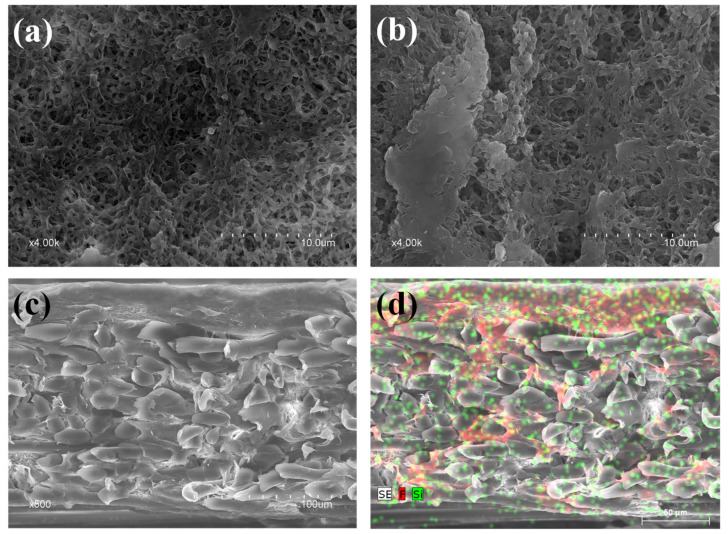
FESEM images of (**a**) non-modified PVDF (nmPVDF) surface, (**b**) modified PVDF (mPVDF_max_) surface, (**c**) modified PVDF (mPVDF_max_) cross-section, and (**d**) EDX mapping of modified PVDF (mPVDF_max_) cross-section (red dots for F and green dots for Si).

An inspection of the surface and cross-section of nmPVDF and mPVDF_max_ was carried out by means of a FESEM analysis. The nmPVDF presented a homogenous surface with a high surface porosity of around 55% (Figure 6a). The effect of membrane modification resulted in a heterogeneous surface with porous zones and zones with dense and smooth clusters (Figure 6b) and less surface porosity of around 27%. Similar observations have been reported by other authors [23,25]. This can be attributed mainly to a coating-like effect of the polymerisation/condensation of the FSi_T_ components (TEOS and Dyn) on the surface leading to a higher hydrophobicity. Thus, these observations led to the conclusion that the homogeneous distribution and availability of the OH^−^ groups on the PVDF chains are critical factors for the grafting and condensation of the FSi_T_ in order to promote a homogeneous surface, since the OH^−^ groups act as active sites [21].

The grafting of the FSi_T_ onto the membrane surface was also verified by measuring the fluorine and silicon content of both nmPVDF and mPVDF_max_ by EDX spectroscopy. The mPVDF_max_ showed a slightly higher F content than that obtained for the nmPVDF (55% and 53%, respectively) indicating that the Dyn had been grafted on the surface. Additionally, the mPVDF_max_ presented a Si content of 0.5% due to the grafting and condensation of the silanols from TEOS over the surface as well as the grafting of Dyn.

From the cross-section inspection, the overall thickness of the mPVDF_max_ was slightly greater than that of the nmPVDF, with values of 164 ± 1 µm and 159 ± 2 µm, respectively. A dense and thick top layer was observed for the mPVDF_max_ with a thickness between 15 and 37 µm (Figure 6c), composed of the residual PVDF chains and especially of the grafted Dyn, since EDX analysis showed a high F content distributed homogeneously throughout this layer (red dots in Figure 6d). An FESEM image of the nmPVDF cross-section is shown in Supplementary Material S4 as an example, and a detailed analysis can be found in our previous work [8]. The Dyn seemed to be grafted both at the surface level and inside the membrane pores, reaching the interface between the PVDF and the PET membrane support and increasing the thickness of that top layer. The presence of this top layer was probably responsible for the increase in hydrophobicity and likely to improve the wetting resistance of the membrane. The EDX analysis also showed the presence of Si (green dots in Figure 6d) from the silica precursor TEOS, located mainly in the top layer, but Si was also found in all the sections but at a lower concentration, as also reported in the literature [41]. Therefore, the condensation of silanols occurred mainly at the surface level [24]. The Si content detected inside the membrane could be attributed to a deposition of the residual FSi_T_ after solvent evaporation in the curing step, since no reactions between silanols and the PET support were expected [21].

Degradation of the mPVDF activated at NaOH%_wt_ levels above 6% was confirmed by FESEM-EDX analyses (Supplementary Material S1 and S4). Circular holes were observed on the membrane surface showing a chemical degradation of the active layer, since no F was detected inside the holes. This degradation was accompanied with a visual colour change of orange stains to the membrane surface. The high concentration of NaOH degraded the membrane by the loss of PVDF chains affecting the membrane integrity [21,31]. The increase in NaOH%_wt_ or activation time increased the extent of defluorination, leading to a severe alteration to the surface chemical structure. For instance, this reaction under severe conditions could convert the PVDF to polyacetylene chains followed by detaching from the surface [24]. Other authors observed a similar colour change in PVDF treated with an alkaline solution, and they established that the F content was lower as the colour became darker [42,43]. A decrease in the mechanical properties of the PVDF after NaOH treatment has been reported, especially for NaOH%_wt_ ≥ 10%, leading to a severe reduction in the degree of crystallinity of the PVDF structure [43]. The PET support could also be degraded, since PET is hydrolysed under severe alkali concentrations and temperature, so the membrane support can play a critical role in the selection of the activation technique and conditions.

### 3.3. Performance of the PVDF Membranes in Stability in Long-Term Operation Tests

**Figure 7:**
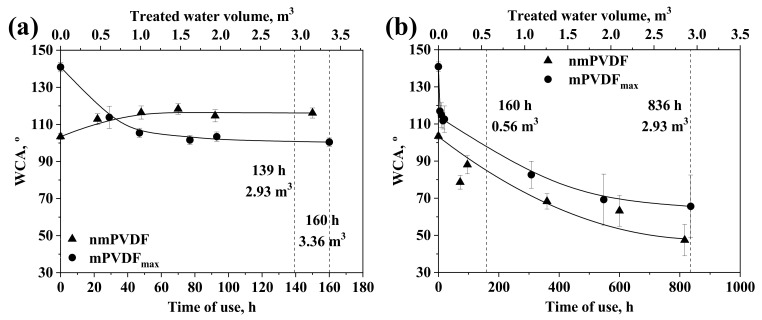
Water contact angle versus time of use (bottom axis) and volume of treated water (top axis) for non-modified PVDF (nmPVDF) and PVDF modified under optimal conditions (mPVDF_max_) with (**a**) deionised water at a liquid flow rate of 21 L h^−1^ and (**b**) anaerobic reactor effluent at a liquid flow rate of 3.5 L h^−1^. Data on nmPVDF with deionised water from [8]. The error bars denote the standard deviation.

The stability of PVDF in long-term operation was evaluated by monitoring its WCA as a useful indicator of changes or alterations occurring on the membrane surface [8,27,33]. The results of the variation in WCA during use of the nmPVDF and mPVDF_max_ are shown in Figure 7a,b for tests with DW and AE, respectively.

The WCA of the mPVDF_max_ decreased continuously from 140.9 ± 2.5° at 0 h to 100.5 ± 2.2° at 160 h in the test with DW (Figure 7a). The WCA decrease was more pronounced in the first 47 h, reaching a value of 105.4 ± 2.5° and then staying almost constant; the final WCA of mPVDF_max_ was similar to the initial value of the nmPVDF (100.5 ± 2.2° and 103.4 ± 1.6°, respectively). The WCA decrease could be due to the reduction of F content detected in the EDX analysis from 55% to 53%, similar to the pristine nmPVDF. This F loss could be attributed to the stress and dragging effect of the liquid flux removing the polymeric chains and/or clusters weakly linked to the surface. Nevertheless, Si content increased up to values of 2% suggesting that the most external layer was composed of the Dyn molecules and the silica precursor was strongly linked to the membrane surface. The WCA after the activation step was of 88.5 ± 5.4°, lower than that of the mPVDF_max_ at 160 h (100.5 ± 2.2°), indicating that siloxane chains and Dyn molecules remained on the surface at the end of the long-term experiment with a moderate liquid flow rate of 21 L h^−1^. Future research would be needed to improve the fixation of macromolecules onto the membrane.

At the end of the long-term test with DW, a permanent plastic deformation at surface level was observed for the mPVDF_max_ from the FESEM images (Supplementary Material S4) in which the surface porosity declined at values lower than 10%. Similar observations were found for the nmPVDF resulting in a dense-like surface [8]. Moreover, the functional top layer formed onto the membrane appeared to have been substantially reduced and the membrane thickness decreased to a value of 148 ± 12 µm, indicating that part of this surface layer was removed.

Some authors have monitored membrane stability and wetting resistance in long-term operations by means of the gas flux change instead of WCA. Using tap water, Sethunga et al. [21] reported a stable CH_4_ flux of around 36 × 10^−5^ g m^−2^ s^−1^ over 240 h at a liquid flow rate of 0.6 L h^−1^ using a modified PVDF, and Wongchitphimon et al. [44] also reported a stable CH_4_ flux of around 66.7 × 10^−5^ g m^−2^ s^−1^ over 300 h at a liquid flow rate of 5.2 L h^−1^ using polyimide membranes functionalised with organofluorosilanes.

Regarding the results with AE at a liquid flow rate of 3.5 L h^−1^ (Figure 7b), the WCA of the nmPVDF decreased continuously from 103.4 ± 1.6° at the beginning to 47.4 ± 8.4° after 816 h, showing a reduction of 54% from the initial value. A gradual darkening of the membrane was apparent during the tests with AE (Supplementary Material S5), indicating that the entire surface was covered by a fouling cake, which could explain the WCA decrease due to the hydrophilisation promoted by some fouling agents [45]. The WCA trend was similar for both the nmPVDF and mPVDF_max_, but the final WCA of the mPVDF_max_ was higher, with a value of 65.6 ± 16.9° after 836 h, representing a reduction of 53% from the initial value (140.9 ± 2.5°) in spite of the mPVDF_max_ surface being darker at the end of the test. In addition, the presence of a fouling cake on the membrane could be confirmed by two additional observations: (1) the WCA initially decreased faster with AE than with DW despite the lower liquid flow rate in both the nmPVDF and mPVDF_max_ membranes (dashed line at 160 h in Figure 7) and (2) the final WCA obtained with AE was lower than with DW at the same volume of treated water and with lower flow rate (dashed line at 2.93 m^3^ of treated water in Figure 7). Regarding observation number (1), the relatively sudden WCA decline of the mPVDF_max_ in the first 50 h could be attributed to a fast attachment of organic/biological matter in AE with more affinity with hydrophobic surfaces [46]. All these results indicated that the membrane surface was covered by the deposition of fouling agents, obscuring the hydrophobic nature of the membrane surface; the hydrophobic level of the surface was dominated by the nature and amount of fouling agents present.

A water breakthrough was detected on the nmPVDF at 816 h, indicating mechanical or chemical deterioration of the membrane surface. Interestingly, a longer useful lifetime was achieved for the mPVDF_max_ since no water breakthrough was observed. Thus, the modification procedure seemed to improve the stability of the PVDF in long-term operations with real AEs. In this regard, the surface modification should be taken into account as a strategy to increase the useful lifetime of the membrane since a greater chemical or mechanical resistance could be induced. In order to compare the viability of using both non-modified and modified membranes, a suitable economic analysis should also consider the additional cost of the modification procedure and its effect on the membrane performance and useful lifetime.

### 3.4. Performance of the PVDF Membranes for the Recovery of Dissolved Methane from Water

The performance of the mPVDF_max_ in degassing tests for D-CH_4_ recovery was evaluated for both DW and AE and compared with the performance of the nmPVDF.

Results obtained with DW are presented in Table 3, in which the D-CH_4_ RE and the methane flux (J_CH4_) at different liquid flow rates (Q_L_) for both materials are compared. An RE of 42% and 21% were obtained for mPVDF_max_ at a Q_L_ of 21.0 and 3.5 L h^−1^, respectively. A slight improvement in membrane performance was achieved after the membrane modification process, especially when a Q_L_ higher than 3.5 L h^−1^ was applied. Thus, the RE at a Q_L_ of 21.0 L h^−1^ was slightly higher than that seen with the nmPVDF (39%). No significant differences were observed in the RE at the lowest Q_L_ of 3.5 L h^−1^ for both membranes, this being attributed mainly to the limiting mass transfer resistance in the liquid phase, especially at low liquid flow rates and velocities [8,37,47].

The CH_4_ flux was also calculated for the degassing tests, and the results are shown in Table 3. The maximum CH_4_ flux was observed for the mPVDF_max_ at the highest Q_L_ of 21 L h^−1^ with a value of 52 × 10^−5^ g s^−1^ m^−2^ since this test showed the maximum RE value. The CH_4_ fluxes obtained in this work under unsteady-state conditions were similar to those reported by other authors operating under steady-state conditions. Wongchitphimon et al. [44] obtained CH_4_ fluxes of 42 × 10^−5^ to 80 × 10^−5^ g s^−1^ m^−2^ for polyimide membranes modified with organofluorosilanes at liquid flow rates ranging from 2.6 to 5.8 L h^−1^. Sethunga et al. [21] reported CH_4_ fluxes of 17 × 10^−5^ to 39 × 10^−5^ g s^−1^ m^−2^ for PVDF modified with organofluorosilanes with Q_L_ ranging from 0.2 to 0.5 L h^−1^. Another work of Sethunga et al. [48] used polydimethylsiloxanes as modifying agents, and CH_4_ fluxes between 16 × 10^−5^ and 48 × 10^−5^ g s^−1^ m^−2^ were reported with Q_L_ ranging from 0.05 to 3.0 L h^−1^. All these abovementioned works conducted degassing tests under steady-state conditions with tap water and initial D-CH_4_ concentrations between 12 and 15 mg L^−1^.

The mPVDF_max_ showed a greater mass transport of CH_4_ than did the nmPVDF under the same liquid and gas hydraulic conditions and at the highest Q_L_ (21.0 L h^−1^), since the obtained RE and CH_4_ flux were higher with the mPVDF_max_. Thus, these results indicated that the mass transfer resistance of the mPVDF_max_ membrane was lower than that of nmPVDF, which could be due to the prevention of membrane wetting associated with the increase in WCA obtained after the modification of the membrane. The lower surface porosity and slightly higher thickness in the mPVDF_max_ seemed not to significantly affect to the overall mass transfer resistance, being that the hydrophobicity increase was the predominant factor in the decrease of the mass transfer resistance. The higher hydrophobicity observed on the surface of the mPVDF_max_ involved an increase in the liquid entry pressure; therefore, the membrane pores were less susceptible to being wetted or filled with the liquid phase. It is well-known that membrane wetting leads to an increase in membrane mass transfer resistance for CH_4_ transport from the liquid phase to the gas phase, since CH_4_ presents a higher diffusion coefficient in air than in water [15,49]. Similarly, an increase in degree of wetting with liquid flow rate has been reported, showing a huge influence on membrane resistance, especially for porous membranes [14]. In addition, the reduction in surface porosity observed after modification can also increase wetting resistance, as previously explained.

Likewise, a RE value of 41% was obtained at a Q_L_ of 21.0 L h^−1^ with mPVDF_max_ after 20 h, when its WCA was reduced to 122°. This RE was similar to the initial value of 42% obtained for the mPVDF_max_ with a WCA of ~140°. Thus, it can be concluded that the modification procedure improved the stability of the PVDF in terms of membrane performance, in contrast to those structural and performance changes previously observed for the nmPVDF [8].

Both the nmPVDF and mPVDF_max_ used in the long-term stability tests (Section 3.3) with AE were evaluated in degassing tests at different times of use in order to determine the effect on D-CH_4_ recovery of an actual stream and level of hydrophobicity. The AE previously saturated with CH_4_ was used as the liquid stream. The results in terms of RE and J_CH4_ at different times of use and at liquid flow rates of 3.5 and 21.0 L h^−1^ for the nmPVDF and mPVDF_max_ are shown in Table 3. Similar performances were obtained at a Q_L_ of 3.5 L h^−1^ for the pristine membranes (time of use of 0 h), with nmPVDF at 720 h and mPVDF_max_ at 528 h, with values of RE and J_CH4_ of around 20% and 26 × 10^−5^ g s^−1^ m^−2^, respectively. Thus, the potential effect on membrane performance of the foulants contained in the liquid stream could be considered negligible for both membranes at low liquid flow rates, at least, for a time of use less than 800 h. In contrast, lower RE values were obtained at the higher Q_L_ of 21.0 L h^−1^ for the used nmPVDF and mPVDF_max_ membranes at 744 and 672 h, respectively. For example, REs of 39% and 34% were obtained for the pristine and used nmPVDF, respectively (decline in J_CH4_ of 17%). This RE decline was more severe for the mPVDF_max_ in that it decreased from 42% to 20% for the pristine and used membrane, respectively, at a liquid flow rate of 21.0 L h^−1^ (decline in J_CH4_ of 51%). In this case, the deposition of foulants onto the membrane involved an additional and non-negligible mass transfer resistance, since the liquid resistance decreased at higher liquid flow rates. Similar declines in CH_4_ flux, and even a severe reduction of up to 90% after only 40 h, have been reported by other authors using modified membranes to treat AEs [10,48,50], while a stable flux was maintained when using tap water [21,44]. The results allowed the conclusion that the modification of PVDF promoted a higher additional mass transfer resistance due to the deposition of foulants contained in the AE hindering D-CH_4_ recovery, even though greater stability and resistance were achieved, since no water breakthrough was observed during the long-term test. The apparent higher fouling deposition on the mPVDF could be due to the increase in membrane hydrophobicity which favoured the deposition of hydrophobic foulants, and/or the change in the surface charge which could promote an increase of the electrostatic attraction between foulants and the modified membrane [46,51]. Further investigations should be focused on the mechanisms and characterisation of fouling in order to propose strategies for its control and prevention.

## 4. Conclusions

The hydrophobicity of a PVDF membrane surface has been maximised by chemical grafting in a three-step procedure (activation, functionalisation, and curing) in order to increase the membrane performance in the recovery of dissolved methane from aqueous streams. The main variables in the modification procedure that significantly affected the water contact angle were found to be the NaOH concentration in the activation step and the modifying agent/precursor (FSi_T_) ratio and concentration in the functionalisation step. The contact angle of the modified PVDF increased continuously with NaOH and FSi_T_ concentration, although the concentration of NaOH was limited by the degradation observed on the membrane surface at concentrations of >5%. In addition, no significant increase in contact angle was observed at an FSi_T_ concentration of >7.2%. In contrast, a maximum contact angle was observed for an FSi_T_ ratio of 0.55. Thus, these values were established as the optimal conditions in order to maximise the hydrophobicity of the membrane surface, achieving a contact angle of >140°. This value represented an increase of >36% with respect to the initial contact angle of the non-modified PVDF (103°).

Regarding the stability of the membrane under long-term operation, the modified PVDF experienced a continuous decrease in contact angle to similar values as the pristine non-modified PVDF in a distilled water stream. In contrast, the contact angle of the modified PVDF was always higher when real anaerobic reactor effluent was used as the liquid stream. However, the final contact angle was quite low, at values of around 70° and 50° for the modified and non-modified PVDF, respectively, after ~800 h. In addition, the non-modified PVDF was strongly degraded at ~800 h since a water breakthrough occurred. Those outcomes allowed the conclusion that the modified PVDF presented greater chemical resistance and stability under operational conditions.

With regard to methane recovery, at the highest liquid flow rate tested (21 L h^−1^), the modified PVDF showed a slightly higher performance than the non-modified PVDF. However, when real AE was used, the performance of the modified PVDF membrane decreased faster, suggesting that modification could favour fouling. In contrast, at the lowest liquid flow rate (3.5 L h^−1^) no significant differences were observed between the modified and non-modified membranes, which could be explained by taking into account the high and limiting mass transfer resistance of the liquid phase under this operational condition, and so the effect of the additional resistances of the membrane could be ignored.

## Figures and Tables

**Table 1:** Characteristics of the flat-sheet PVDF membrane.

Property	Value
Structure	Microporous
Support	Polyester (PET)
Thickness, µm	159 ± 2 ^a^
Pore diameter, µm	0.2 ^b^
Porosity, %	62 ± 3 ^c^
Liquid entry pressure, bar	1.8 ^b^
Static water contact angle, °	103.4 ± 1.6 ^a^

^a^ Measured. ^b^ Provided by the manufacturer (Dorsan Filtration, Spain). ^c^ Determined by gravimetric analysis [32].

**Table 2:** Factors and levels used in the design of the experiments and statistical analysis of the modification procedure.

Independent Variables (Factors)		Levels				
		Axis (−)	Corner (−)	Central Point	Corner (+)	Axis (+)
A	FSi_T_ ratio	0.17	0.29	0.55	0.79	0.94
B	FSi_T_%_v_, %	0.8	2.4	4.6	7.2	9.2
C	NaOH%_wt_, %	1.0	2.0	3.5	5.0	6.0

**Table 3:** Effect of the time of use on the dissolved CH_4_ removal efficiency (RE) and CH_4_ flux (J_CH4_) in degassing tests (at 5 h of time on stream) with non-modified PVDF (nmPVDF) and modified PVDF (mPVDF_max_) at different liquid flow rates (Q_L_) of distilled water and anaerobic reactor effluent.

Membrane	Q_L_, L h^−1^	Time of Use, h	RE, %	J_CH4_ × 10^5^, g s^−1^ m^−2^
(a) With deionised water				
nmPVDF	3.5	0	19 ± 1 ^a^	27 ^a^
	21.0	0	39 ± 1 ^a^	46 ^a^
mPVDF_max_	3.5	0	21 ± 2	29
	21.0	0	42 ± 1	52
(b) With anaerobic effluent				
nmPVDF	3.5	0	19 ± 1	27
	3.5	720	19 ± 2	26
	21.0	0	39 ± 1	46
	21.0	744	34 ± 2	39
mPVDF_max_	3.5	0	22 ± 3	29
	3.5	528	19 ± 1	24
	21.0	0	42 ± 1	52
	21.0	672	20 ± 1	26

^a^ Previous work [8].

## Data Availability

The data presented in this study are available on request from the corresponding author.

## Supplementary Materials

The following supporting information can be downloaded at: https://www.mdpi.com/article/10.3390/membranes12040426/s1: Figure S1. (a) Effect of the NaOH concentration (NaOH%_wt_) (activation time of 1 h at 60 °C) and (b) the activation time (5% NaOH at 50 °C) on the water contact angle (WCA) after activation step. Table S1. Effect of the composition of the functionalisation solution on the water contact angle (WCA) after the curing step of the modified PVDF (activation: 5% NaOH, 1 h, 50 °C; functionalisation: 1 h, room temperature; curing: overnight, 60 °C), Figure S2. Effect of the functionalisation time on the water contact angle (WCA) after the curing step (activation: 5% NaOH, 50 °C, 1 h; functionalisation: FSi_T_%_v_ = 3.6%, FSi_T_ ratio = 0.41; curing: overnight, 60 °C), Figure S3. Effect of the curing time on water contact angle (WCA) after the curing step (activation: 5% NaOH, 50 °C, 1 h; functionalisation: FSi_T_%_v_ = 3.6%, FSi_T_ ratio = 0.41, 1 h; curing: 60 °C), Figure S4. Hydroxylation reaction of the poly(vinylidene fluoride) (PVDF) with sodium hydroxide in the activation step, Figure S5. Hydrolysis reaction of the tetraethyl orthosilicate (TEOS), Figure S6. Condensation reaction of the silanols on the hydroxylated PVDF in the functionalisation step, Figure S7. Condensation reaction of the 1H,1H,2H,2H-perfluorooctyltriethoxysilane (Dynasylan F8261®) onto the membrane surface in the functionalisation step, Figure S8. Examples of the profiles of (a) dissolved methane concentration (C_L_) and removal efficiency (RE) and (b) CH_4_ flux (J_CH4_) versus time on stream for degassing tests with the modified PVDF with the highest hydrophobicity (mPVDF_max_) at a liquid and N_2_ flow rate of 21 L h^1^ and 4 L h^−1^, respectively, Figure S9. Cross section FESEM image of the non-modified PVDF, Figure S10. (a) FESEM image of a modified PVDF surface focused on a degraded zone (membrane activated with a 50%_wt_ NaOH solution). (b) Fluor distribution on the membrane surface around a degraded zone, Figure S11. FESEM images of the (a) surface and (b) cross section of the modified PVDF (mPVDF_max_) surface after 160 h of use with de-ionized water at a liquid flow rate of 21 L h^−1^, Figure S12. Images of the non-modified and modified PVDF membrane surface (nmPVDF and mPVDF_max_, respectively) during the long-term tests with an anaerobic reactor effluent at a liquid flow rate of 3.5 L h^−1^. References [23,24,25,27,29,30,31,33,34,42,43] are cited in supplementary materials.
