# Supplementary material for: Flat PVDF Membrane with Enhanced Hydrophobicity through Alkali Activation and Organofluorosilanisation for Dissolved Methane Recovery
Source: Membranes (Basel). 2022 Apr 15;12(4):426. doi: 10.3390/membranes12040426 (PMC9027404; doi:10.3390/membranes12040426)
Supplement: Supplementary file 1 [file membranes-12-00426-s001.zip › membranes-1632775-supplementary.pdf]

membranes

MDPI

Supplementary Material

Flat PVDF membrane with enhanced hydrophobicity through alkali activation and organofluorosilanisation for dissolved methane recovery

Ramón Jiménez-Robles [1], Beatriz María Moreno-Torralbo [2], Jose David Badia [2], Vicente Martínez-Soria [1] and Marta Izquierdo [1,*]

[1] Research Group in Environmental Engineering (GI²AM), Department of Chemical Engineering, School of Engineering, University of Valencia, Avda. Universitat s/n, 46100 Burjassot, Spain; ramon.jimenez@uv.es (R.J.-R.); vmsoria@uv.es (V.M.-S.)
[2] Research Group in Materials Technology and Sustainability (MATS), Department of Chemical Engineering, School of Engineering, University of Valencia, Avda. Universitat s/n, 46100 Burjassot, Spain; bmmortor@itq.upv.es (B.M.M.-T.); jose.badia@uv.es (J.D.B.)
* Correspondence: marta.izquierdo-sanchis@uv.es; Tel.: +34-963-543-737; Fax: +34-963-544-898

S1. Preliminary experiments: effect on surface hydrophobicity of activation, functionalisation and curing time, and solutions composition.

Several preliminary experiments were carried out in order to stablish the suitable range of the different parameters involved in the surface modification process that affected the membrane hydrophobicity. The suitable values determined for those parameters were used for the design of experiments to maximize the static water contact angle (WCA) of the modified PVDF.

The NaOH concentration (NaOH$\%_{wt}$) applied in the activation step and the activation time were evaluated, with NaOH weight concentrations ranging from 0.04% to 50% (**Error! Reference source not found.**a). The WCA of the membrane after activation decreased continuously with the increase of NaOH$\%_{wt}$ due to a higher extent of the defluorination process of the PVDF as reported by [1,2] with a less hydrophobic surface. The degradation on the membrane surface was observed since a colour change was detected on the membranes activated at NaOH$\%_{wt}$ higher than 6% appearing orange stains in a heterogeneous way. FESEM images confirmed this degradation in the form of holes for NaOH$\%_{wt}$ ≥ 10% mainly due to the severe degradation of the PVDF chains and the PET support of the membrane in a concentrated alkali solution [3] (Supplementary Material S4). Thus, NaOH$\%_{wt}$ was kept in values lower than or equal to 6%.

Regarding the activation time (**Error! Reference source not found.**b), no significant changes were observed in the WCA after 1 h, and important degradation on the membrane surface with FESEM images was observed at 2 hours of activation. Thus, an activation time of 1 h was stablished for all the following experiments. This result was in accordance with that reported by Ross et al. [4], who determined that the most of the deflourination/hydroxylation reaction occurred within the first hour with a F content decrease from a 45.0% to a 25.5%, and stayed in values of 24.4 and 23.6% at 2 and 4 h, respectively. Zheng et al. [1] also reported a clear decrease of the WCA of the PVDF treated at a higher NaOH$\%_{wt}$ of 30% with an activation time of 1 h.

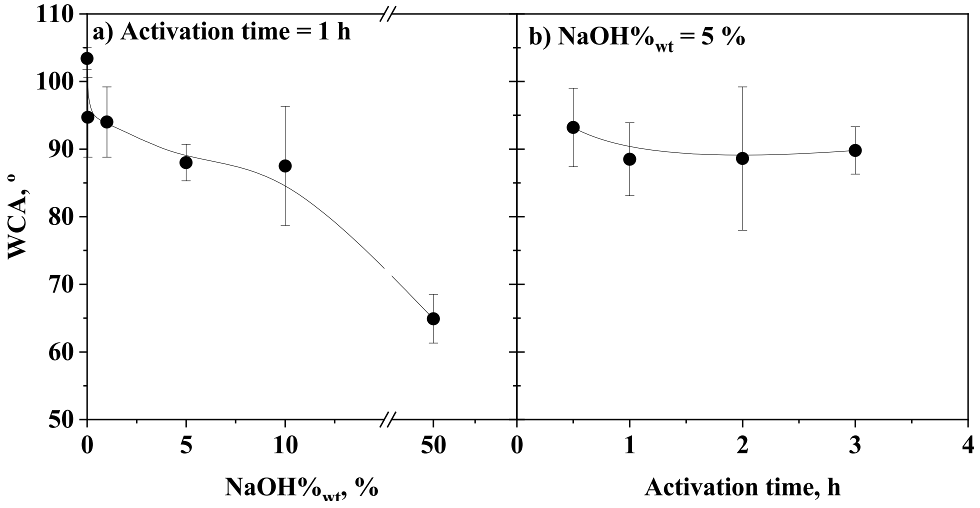

Figure S1. a) Effect of the NaOH concentration ($NaOH\%_{wt}$) (activation time of 1 h at 60ºC) and b) the activation time (5% NaOH at 50ºC) on the water contact angle (WCA) after activation step.

An initial mixture of Dynasylan F8261® (Dyn) and TEOS for the preparation of the functionalisation solution is labelled as FSi_T, where "F" denotes the fluorine modifying agent Dynasylan F8261® and "Si_T" the silica precursor TEOS. Different parameters involved in the functionalisation step were also evaluated (**Error! Reference source not found.**). Regarding the volumetric ratio between the Dyn and TEOS in the functionalisation solution (FSi_T ratio = Dyn volume / FSi_T mixture volume), the WCA showed similar values (~130º) from a FSi_T ratio of 0.41 to 0.74 (**Error! Reference source not found.**, exp. b – d), with a FSi_T percentage in the functionalisation solution ($FSi_T\%_v$) of 3.6%. Also, the WCA did not increase when $FSi_T\%_v$ was increased from 3.6% to 15.1% (**Error! Reference source not found.**, exp. d - e). In addition, other authors reported a WCA decrease for an organosilanes concentration higher than 20% due to the self-condensation of the silanols groups [1]. Thus, the optimum values of FSi_T ratio and $FSi_T\%_v$ that maximize the WCA of the modified PVDF were expected to be found in those ranges.

Table S1. Effect of the composition of the functionalisation solution on the water contact angle (WCA) after the curing step of the modified PVDF (activation: 5% NaOH, 1 h, 50 ºC; functionalisation: 1 h, room temperature; curing: overnight, 60ºC).

Exp.	FSi_T ratio	$FSi_T\%_v$ %	IPA/H_2O ratio mol:mol	WCA º
**a**	0.17	3.6	57:1	120.4 ± 5.1
**b**	0.41	3.6	57:1	131.6 ± 4.0
**c**	0.52	3.6	57:1	129.8 ± 3.8
**d**	0.74	3.6	57:1	134.0 ± 4.7
**e**	0.74	15.1	57:1	129.6 ± 4.4

FSi_T ratio: volumetric ratio between the modifying agent and the silica precursor (Dyn volume / FSi_T mixture volume).
$FSi_T\%_v$: volumetric percentage of the FSi_T mixture in the functionalization solution.
IPA: 2-propanol.

Regarding the functionalisation time, Sairiam et al. [2] reported a WCA increase from 69º to around 85º from 0 to 6 h using hexane as solvent for the functionalisation solution and the same fluorine modifying agent (1H,1H,2H,2H-perfluorooctyltriethoxysilane). In contrast, in our experiments with a mixture of IPA/H_2O as solvent, the WCA remained constant after 1 h (**Error! Reference source not found.**), so a functionalisation time of 1 h was stablished for the experiments.

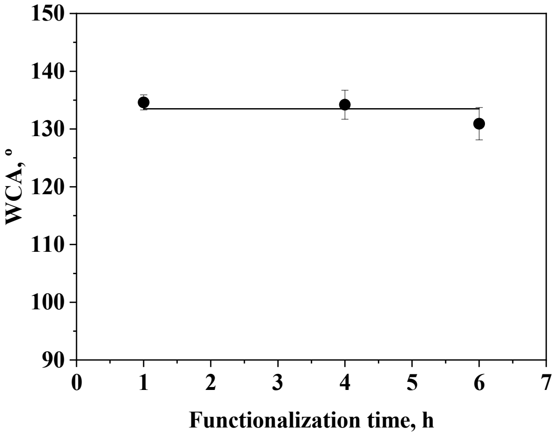

Figure S2. Effect of the functionalisation time on the water contact angle (WCA) after the curing step (activation: 5% NaOH, 50ºC, 1 h; functionalisation: $FSi_T\%_v$ = 3.6%, FSi_T ratio = 0.41; curing: overnight, 60ºC).

The duration of the curing step was also evaluated. The WCA initially increased with the curing time and it remained constant from 7 h (**Error! Reference source not found.**). Thus, a curing time of at least 7 h was applied for the modification procedure of the PVDF.

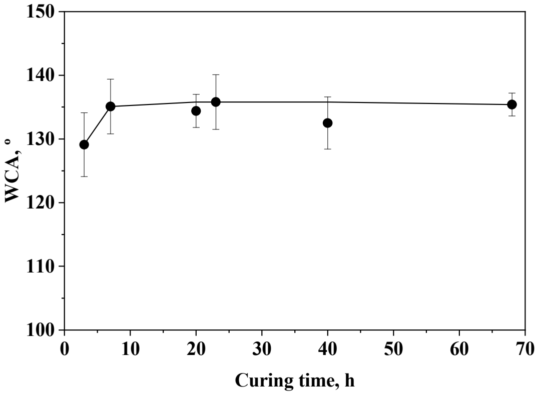

Figure S3. Effect of the curing time on water contact angle (WCA) after the curing step (activation: 5% NaOH, 50ºC, 1 h; functionalisation: $FSi_T\%_v$ = 3.6%, FSi_T ratio = 0.41, 1 h; curing: 60ºC).

S2. Chemical reactions in the activation, functionalisation and curing steps

The surface chemical modification of poly(vinylidene fluoride) (PVDF) consisted in a three-step procedure: 1) activation, 2) functionalisation and 3) curing. In the activation step, PVDF was immersed in a NaOH solution in order to substitute the F atoms from the fluorine chains by OH^- [3–5]. This reaction is called hydroxylation and is shown in **Error! Reference source not found.**. Secondary reactions could occurred involving the formation of carbonyl (C=O) when severe conditions of the activation step, i.e. high temperature, time and NaOH concentration, are used or when a not homogeneous NaOH attack is produced [4]. C-O-F groups can also be formed since the total defluorination does not occur [4].

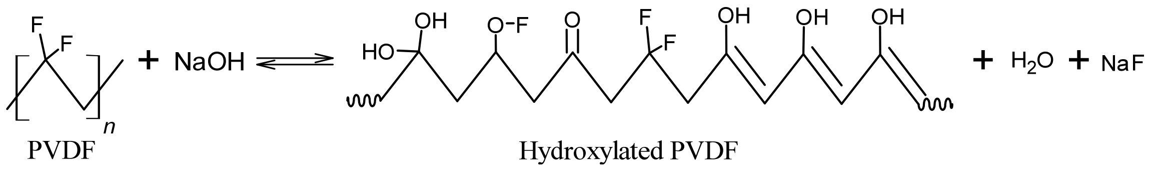

Figure S4. Hydroxylation reaction of the poly(vinylidene fluoride) (PVDF) with sodium hydroxide in the activation step.

For the functionalisation step, the ethoxy groups of the tetraethyl orthosilicate (TEOS) were hydrolysed to silanols during the preparation of the functionalisation solution [6–9] (**Error! Reference source not found.**). The silanol molecules coordinate with the hydroxyl group on the activated PVDF grafting the silica precursor [2,7]. In addition, silanols are chemically reactive and they can condensate forming oxane bonds between Si atoms [2,10,11]. Thus, the proposed chemical reaction and structure is shown in **Error! Reference source not found.**.

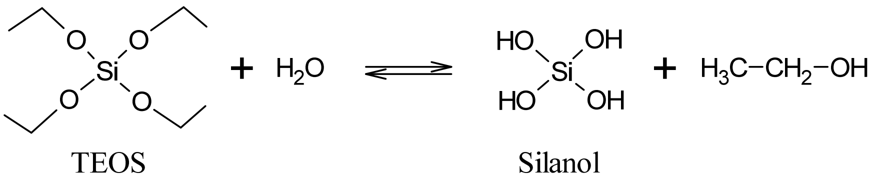

Figure S5. Hydrolysis reaction of the tetraethyl orthosilicate (TEOS).

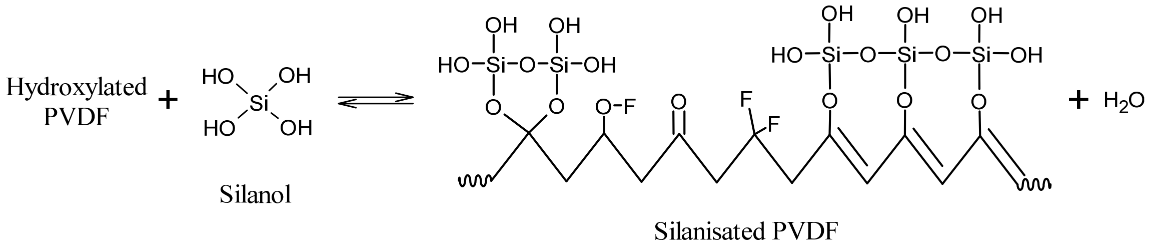

Figure S6. Condensation reaction of the silanols on the hydroxylated PVDF in the functionalisation step.

Finally, the organofluorosilane 1H,1H,2H,2H-perfluorooctyltriethoxysilane (Dynasylan F8261®) as modifying agent is grafted on the membrane by coordinating with the silanols groups [7], as proposed in **Error! Reference source not found.**. The secondary reaction involved in the activation step, the residual fluorine and the non-homogeneous condensation of the silanols can lead to a heterogeneous surface.

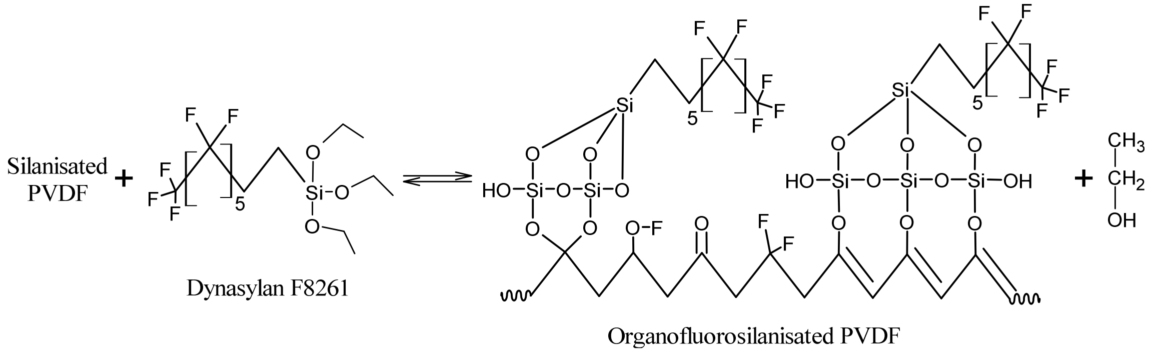

Figure S7. Condensation reaction of the 1H,1H,2H,2H-perfluorooctyltriethoxysilane (Dynasylan F8261®) onto the membrane surface in the functionalisation step.

S3. Dissolved methane concentration, removal efficiency and methane flux profiles

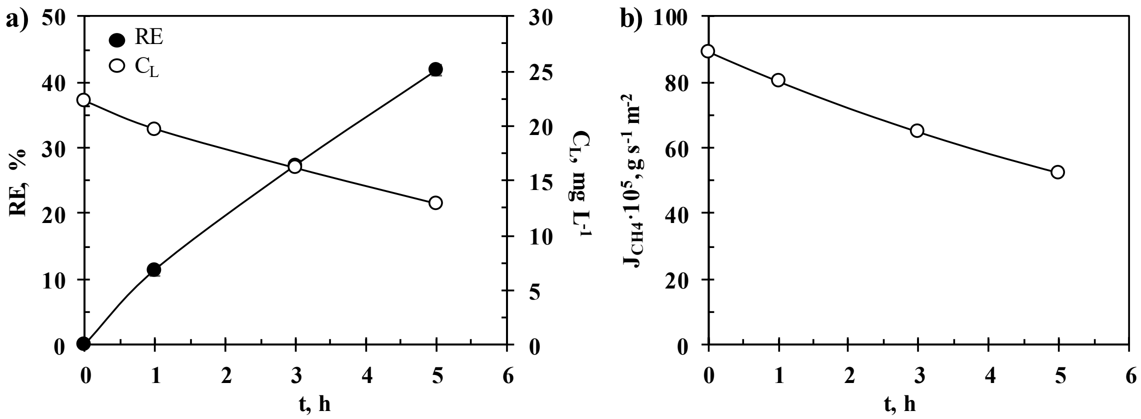

Figure S8. Examples of the profiles of a) dissolved methane concentration (C_L) and removal efficiency (RE) and b) CH_4 flux (J_{CH4}) versus time on stream for degassing tests with the modified PVDF with the highest hydrophobicity ($mPVDF_{max}$) at a liquid and N_2 flow rate of 21 L h^{1} and 4 L h^{-1}, respectively.

S4. Microscopy and elemental analysis on PVDF membranes

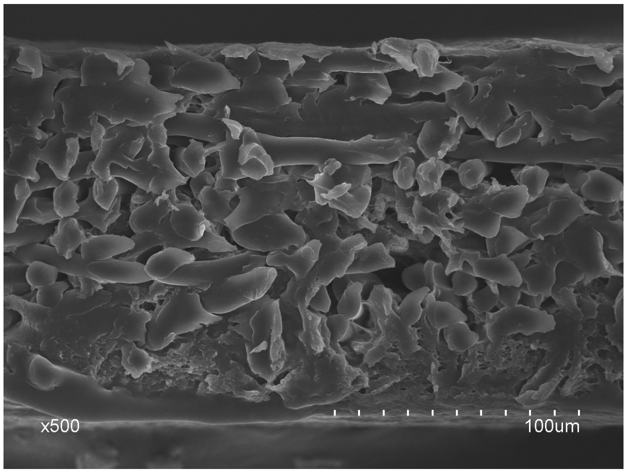

Figure S9. Cross section FESEM image of the non-modified PVDF.

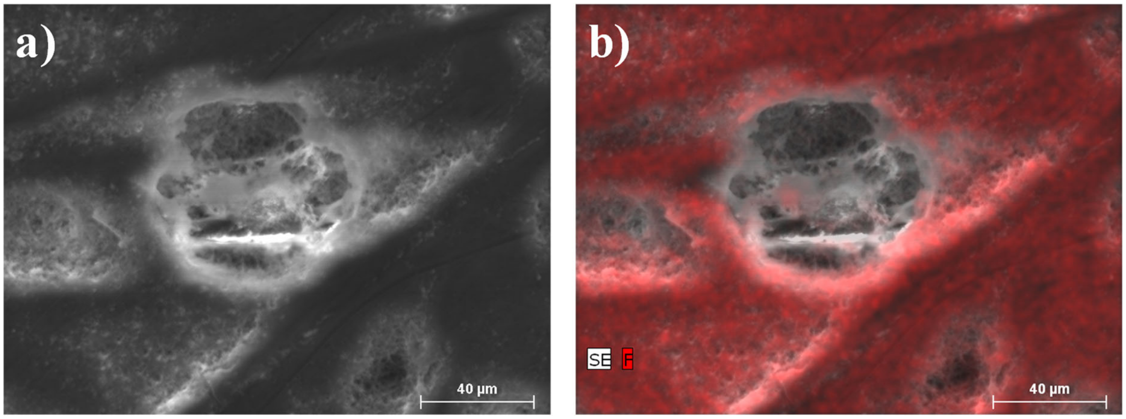

Figure S10. a) FESEM image of a modified PVDF surface focused on a degraded zone (membrane activated with a 50%$_{wt}$ NaOH solution). b) Fluor distribution on the membrane surface around a degraded zone.

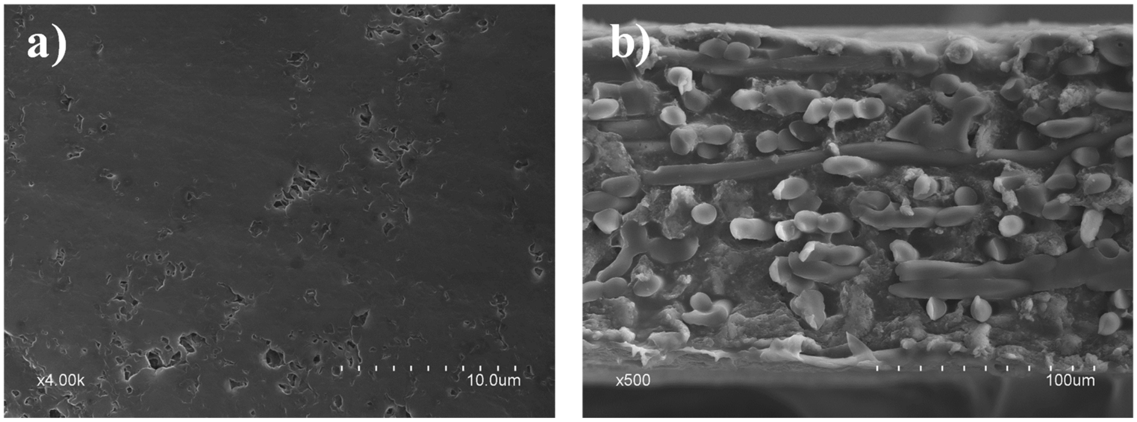

Figure S11. FESEM images of the a) surface and b) cross section of the modified PVDF (mPVDF$_{max}$) surface after 160 h of use with de-ionized water at a liquid flow rate of 21 L h^{-1}.

S5. Images of the membrane surfaces during the long-term tests with an anaerobic reactor effluent

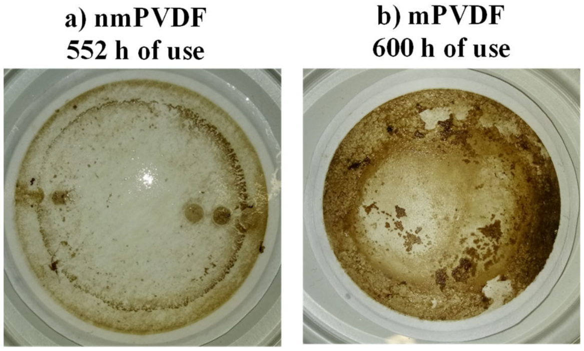

Figure S12. Images of the non-modified and modified PVDF membrane surface (nmPVDF and mPVDF$_{max}$, respectively) during the long-term tests with an anaerobic reactor effluent at a liquid flow rate of 3.5 L h^{-1}.

References

1. Zheng, Z.; Gu, Z.; Huo, R.; Luo, Z. Superhydrophobic poly(vinylidene fluoride) film fabricated by alkali treatment enhancing chemical bath deposition. *Appl. Surf. Sci.* **2009**, *256*, 2061–2065, https://doi.org/10.1016/j.apsusc.2009.09.048.
2. Sairiam, S.; Loh, C.H.; Wang, R.; Jiraratananon, R. Surface modification of PVDF hollow fiber membrane to enhance hydrophobicity using organosilanes. *J. Appl. Polym. Sci.* **2013**, *130*, 610–621, https://doi.org/10.1002/app.39197.
3. Hashim, N.A.; Liu, Y.; Li, K. Stability of PVDF hollow fibre membranes in sodium hydroxide aqueous solution. *Chem. Eng. Sci.* **2011**, *66*, 1565–1575, https://doi.org/10.1016/j.ces.2010.12.019.
4. Ross, G.; Watts, J.; Hill, M.; Morrissey, P. Surface modification of poly(vinylidene fluoride) by alkaline treatment1. The degradation mechanism. *Polymer* **2000**, *41*, 1685–1696, https://doi.org/10.1016/s0032-3861(99)00343-2.
5. Ross, G.; Watts, J.; Hill, M.; Morrissey, P. Surface modification of poly(vinylidene fluoride) by alkaline treatment Part 2. Process modification by the use of phase transfer catalysts. *Polymer* **2001**, *42*, 403–413, https://doi.org/10.1016/s0032-3861(00)00328-1.
6. Fabbri, P.; Messori, M.; Montecchi, M.; Pilati, F.; Taurino, R.; Tonelli, C.; Toselli, M. Surface properties of fluorinated hybrid coatings. *J. Appl. Polym. Sci.* **2006**, *102*, 1483–1488, https://doi.org/10.1002/app.24350.
7. Wongchitphimon, S.; Wang, R.; Jiraratananon, R. Surface modification of polyvinylidene fluoride-co-hexafluoropropylene (PVDF–HFP) hollow fiber membrane for membrane gas absorption. *J. Membr. Sci.* **2011**, *381*, 183–191, https://doi.org/10.1016/j.memsci.2011.07.022.
8. Oldani, V.; Sergi, G.; Pirola, C.; Sacchi, B.; Bianchi, C. Sol-gel hybrid coatings containing silica and a perfluoropolyether derivative with high resistance and anti-fouling properties in liquid media. *J. Fluor. Chem.* **2016**, *188*, 43–49, https://doi.org/10.1016/j.jfluchem.2016.06.005.
9. Huang, X.; Zhang, J.; Wang, W.; Liu, Y.; Zhang, Z.; Li, L.; Fan, W. Effects of PVDF/SiO2 hybrid ultrafiltration membranes by sol–gel method for the concentration of fennel oil in herbal water extract. *RSC Adv.* **2015**, *5*, 18258–18266, https://doi.org/10.1039/c4ra15448g.
10. Zhang, Y.; Wang, R. Fabrication of novel polyetherimide-fluorinated silica organic–inorganic composite hollow fiber membranes intended for membrane contactor application. *J. Membr. Sci.* **2013**, *443*, 170–180, https://doi.org/10.1016/j.memsci.2013.04.062.
11. Zhang, Y.; Wang, R. Novel method for incorporating hydrophobic silica nanoparticles on polyetherimide hollow fiber membranes for CO2 absorption in a gas–liquid membrane contactor. *J. Membr. Sci.* **2013**, *452*, 379–389, https://doi.org/10.1016/j.memsci.2013.10.011.
